# P-505. Geospatial Patterns of Maternal Syphilis in Northern Mexico: An 8-Year Data Analysis

**DOI:** 10.1093/ofid/ofaf695.720

**Published:** 2026-01-11

**Authors:** Paola Quintanilla-Urdiales, Abril M Gutiérrez-Castro, Rubén G Valadez-Mata, Ian Carlo Pineda-Fierro, Judith Estela Guzman Garcia, Jessica Guerra-Díaz, Rocio Ximena Sandoval-Orozco, Oscar Tamez-Rivera, Lindsay Ariadna Concha-Mora

**Affiliations:** Pediatric Residency Program, Programa Multicéntrico de Especialidades Médicas ITESM- SSNL, Tecnológico de Monterrey. Escuela de Medicina y Ciencias de la Salud. Monterrey, México, Monterrey, Nuevo Leon, Mexico; Pediatric Residency Program, Programa Multicéntrico de Especialidades Médicas ITESM- SSNL, Tecnológico de Monterrey. Escuela de Medicina y Ciencias de la Salud. Monterrey, México, Monterrey, Nuevo Leon, Mexico; Hospital Universitario Dr. Jose Eleuterio Gonzalez, Guadalupe, Nuevo Leon, Mexico; Hospital Universitario Dr. Jose Eleuterio Gonzalez, Guadalupe, Nuevo Leon, Mexico; Tecnologico de Monterrey, Monterrey, Nuevo Leon, Mexico; Hospital Universitario Dr. José Eleuterio González, Monterrey, Nuevo Leon, Mexico; Tecnológico de Monterrey Campus Monterrey, Chihuahua, Chihuahua, Mexico; Tecnologico de Monterrey, Escuela de Medicina y Ciencias de la Salud, Monterrey, Nuevo Leon, Mexico; The Hospital for Sick Children, Toronto, ON, Canada

## Abstract

**Background:**

Syphilis remains a major public health issue. In 2024, the WHO estimated that 8 million adults acquired syphilis worldwide, and >700,000 cases of congenital syphilis were reported. In Mexico, maternal syphilis rose by 222% between 2016 - 2022, highlighting the need for enhanced surveillance strategies. Geographic Information Systems (GIS) offer valuable information for public health interventions. Previous studies have consistently demonstrated that distances >10 km from a facility with diagnostic and therapeutic capacity negatively impact patient outcomes.

**Figure:**
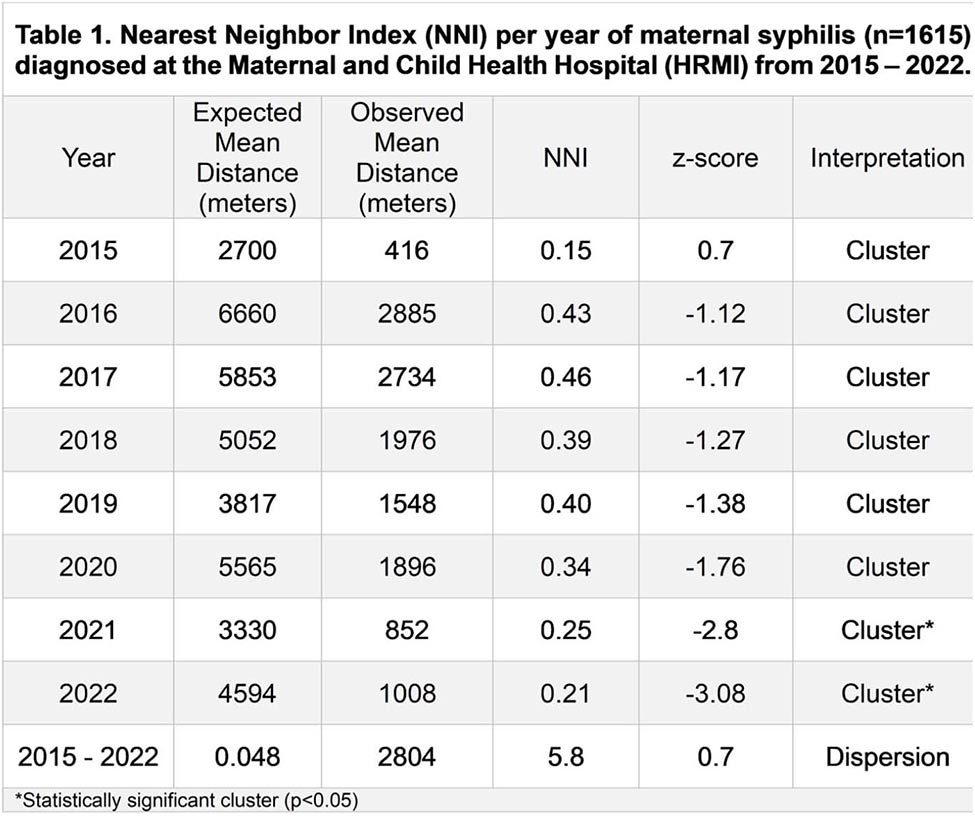

**Image 1.**
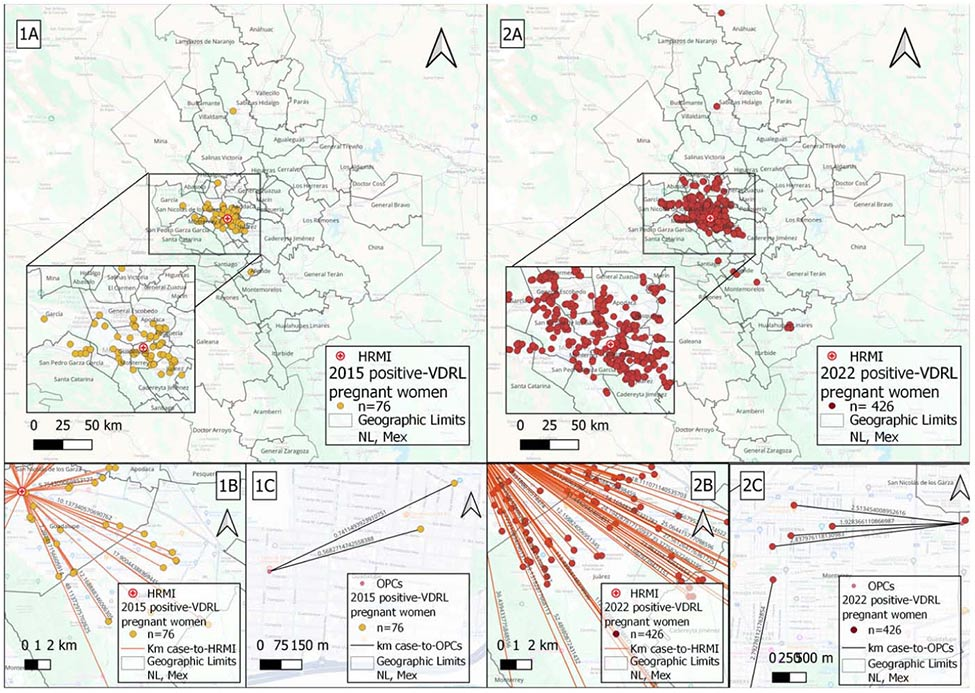
Distribution and distance analysis of VDRL-positive pregnant women diagnosed at the Maternal and Child Health Hospital (HRMI) from 2015 – 2022. 1A. Geographic distribution of VDRL-pregnant women diagnosed at the HRMI in 2015. 1B. Distance in km between case-to-HRMI of 2015. 1C. Distance in km between case-to-nearest OPCs of 2015. 2A Geographic distribution of VDRL-pregnant women diagnosed at the HRMI in 2022. 1B. Distance in km between case-to-HRMI of 2022. 1C. Distance in km between case-to-nearest OPCs of 2022.

**Methods:**

We analyzed the records of pregnant women with a positive VDRL test and their newborns treated at the reference Maternal and Child Health Hospital (HRMI) in NL, Mexico, between 2015 and 2022. Geolocation was performed using complete residential addresses to generate X and Y coordinates in QGIS®. Spatial analysis included the Nearest Neighbor Index (NNI), hierarchical clustering, and kernel density estimation to assess distribution and proximity between cases, outpatient clinics (OPC), and HRMI.

**Image 2.**
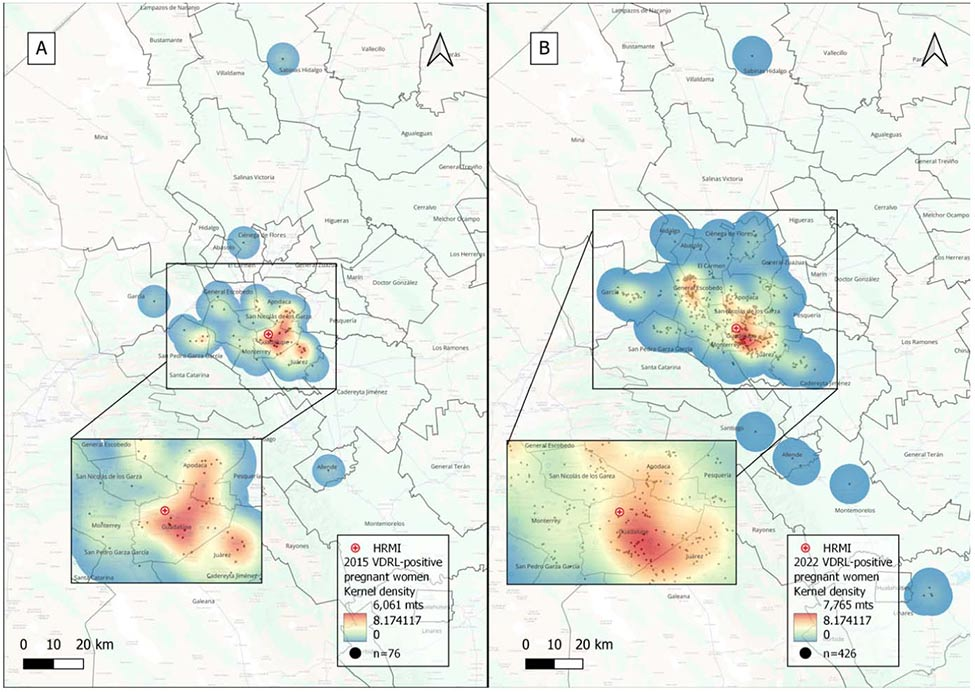
Heat Maps of VDRL-positive pregnant women diagnosed at the Maternal and Child Health Hospital (HRMI) from 2015 – 2022. A. Heat Map of VDRL-pregnant women diagnosed at the HRMI in 2015 with a KDE of 6,061 mts B. Heat Map of VDRL-pregnant women diagnosed at the HRMI in 2022 with a KDE of 7,765 mts

**Results:**

Data from 1615 VDRL-positive pregnant women at the time of delivery were included. Cases were heavily concentrated in 3 urban municipalities. Mean distance from cases to the nearest OPC was 0.8 km and 22.5 km to HRMI. Most (67%) pregnant women lived >10 km from HRMI. In contrast, 79% lived within 2 km of an OPC, where diagnosis was often missed. NNI revealed a dispersive pattern; however, a temporal trend toward clustering was observed, reaching statistical significance in 2021 when cases began forming significant clusters (p < 0.05).

**Conclusion:**

We identified hotspots of maternal syphilis in NL Mexico, revealing a mismatch between the location of high-burden areas and access to diagnostic and treatment services. The increasing spatial clustering over time, alongside the limited diagnostic capacity of OPCs, highlights systemic gaps in timely detection and care. Our findings emphasize the need to decentralize syphilis management by establishing specialized maternal care services closer to affected communities and offering healthcare personnel training to existing OPCs. These geospatial insights offer a critical foundation for targeted public health interventions.

**Disclosures:**

All Authors: No reported disclosures

